# Two Divergent Isolates of Turnip Yellows Virus from Pea and Rapeseed and First Report of Turnip Yellows Virus-Associated RNA in Germany

**DOI:** 10.1128/MRA.00214-19

**Published:** 2019-04-25

**Authors:** Yahya Z. A. Gaafar, Heiko Ziebell

**Affiliations:** a Institute for Epidemiology and Pathogen Diagnostics, Julius Kühn-Institut, Braunschweig, Germany; Queens College

## Abstract

Two divergent isolates of turnip yellows virus (TuYV) were identified in pea and rapeseed. The nearly complete genome sequences of the virus isolates share 93.3% nucleotide identity with each other and 89.7% and 92.9% with their closest isolate from South Africa. Additionally, a turnip yellows virus-associated RNA was identified.

## Abstract (precis)

Two divergent isolates of turnip yellows virus (TuYV) were identified in pea and rapeseed. The nearly complete genome sequences of the virus isolates share 93.3% nucleotide identity with each other and 89.7% and 92.9% with their closest isolate from South Africa.

## ANNOUNCEMENT

Turnip yellows virus (TuYV), the non-sugar beet-infecting strain of beet western yellows virus (BWYV), is a polerovirus (family *Luteoviridae*) (1, 2). TuYV can infect a wide range of crops, predominantly members of the *Brassicaceae* and *Fabaceae* families.

Two plant samples (pea [Pisum sativum] and oilseed rape [Brassica napus]) displaying yellowing symptoms were collected in Germany (in 2016 and 2006, respectively). The samples were tested with a triple antibody sandwich enzyme-linked immunosorbent assay (TAS-ELISA) for the presence of luteoviruses/poleroviruses using monoclonal antibodies 5G4 and 6G4 (3), as described (4, 5). Both samples tested positive for polerovirus infection but showed different titers, which prompted us to determine the genomic sequences of these isolates. The viruses were maintained on pea (isolate identifier [ID] JKI 29344) and radish (JKI 29345) by aphid transmission using Myzus persicae. Nonviruliferous aphids were left to feed for 3 days on the infected leaves, and then 10 aphids were transferred to healthy plants for 3 days (3 cycles for the pea isolate and 33 cycles for the oilseed rape isolate). Four weeks post-aphid inoculation, polerovirus infection of plants was confirmed using TAS-ELISA and reverse transcription-PCR (RT-PCR) with generic polerovirus primers (S2 and AS3) (6). The RT-PCR bands were Sanger sequenced, and a BLASTn search resulted in the highest hits, with 99% (pea isolate) and 100% (oilseed rape isolate) nucleotide identities to the partial coat protein sequences of BWYV (GenBank accession number L39976) and TuYV (GenBank accession number KU198395).

For genome sequencing, total RNAs were extracted with the innuPREP RNA minikit (Analytik Jena AG), followed by rRNA depletion with the RiboMinus plant kit (Invitrogen). cDNAs were synthesized using ProtoScript II reverse transcriptase (NEB) and random octanucleotide primers, followed by second-strand synthesis with the NEBNext Ultra II nondirectional RNA second-strand synthesis module kit (NEB). The libraries were prepared using a Nextera XT library kit (Illumina) and submitted for high-throughput sequencing (HTS) on the MiSeq version 3 platform (2 × 301). The raw reads (total reads, 1,640,360 for JKI 29344 and 1,648,784 for JKI 29345) were analyzed using the Geneious software (11.1.4). The reads were quality trimmed (error limit, 0.05) and size filtered to >99 nucleotides (nt), followed by *de novo* assembly using the Geneious assembler (parameter, medium sensitivity/fast). The assembled contigs were used to search the NCBI database using BLASTn. A contig of about 5.6 kb in each sample showed 90.7% and 93.1% nt identities to TuYV (GenBank accession number KU198395). Contig extension using Geneious mapping to the reference tool (parameter, medium sensitivity/fast) resulted in the complete coding sequences and almost-full-genomic sequences for both TuYV isolates; they shared 93.3% nt identity to each other while sharing 89.7% and 92.9%, respectively, to the most closely related isolate, KU198395.

Pairwise comparisons of the amino acid sequences using MUSCLE (3.8.425) showed that some open reading frames (ORFs) are also highly divergent (P0 and P1), whereas others are not (CP and MP) (Table 1) (7). The pea and the oilseed rape isolates’ ORFs shared between 80.9% and 99.5% amino acid (aa) identities to each other and between 74.1% and 95.6% in comparison to their homologues of KU198395.

**TABLE 1:** Pairwise amino acid comparisons between the predicted proteins of the German TuYV isolates and their homologues of KU198395 using MUSCLE 3.8.425

Protein source (isolate)	Amino acid identity (%) by open reading frame(s)						Reference isolate or accession no.
	P0	P1-P2	P1	P3-P5	CP	MP	
Pea (JKI 29344)	80.9	90.6	86.5	97.8	99.5	99.4	JKI 29345
	74.1	89.8	85.3	94.1	93.1	90.9	KU198395
Oilseed rape (JKI 29345)	85.9	95.6	94.2	93.7	92.6	90.3	KU198395

An additional contig of about 2.8 kb was found in the oilseed rape sample that shared 98% nt identity with the partial sequence of beet western yellows virus-associated RNA (BWYVaRNA) from the United Kingdom (GenBank accession number KF533709) (8). Polerovirus-associated RNAs are single-stranded RNAs (ssRNAs) of ∼2.8 to 3 kb and have two major ORFs. They replicate autonomously and appear to depend on a helper virus for aphid transmission by encapsidating within the virus coat protein (9). They may increase the severity of disease symptoms. The full genome of 2,841 nt was assembled by mapping to the reference sequence with NCBI RefSeq accession number NC_004045 (10), and we propose the name “turnip yellows virus-associated RNA” (TuYVaRNA) for this RNA. While TuYVaRNA shares 93% nucleotide identity with NC_004045 and 98% with the partial sequence from the United Kingdom, its genomic organization was similar to that of the other polerovirus-associated RNAs, containing three ORFs, with the first one containing an amber readthrough ORF.

To our knowledge, these are the first complete coding sequences of TuYV and the first report of TuYVaRNA from Germany.

### Data availability.

The complete coding sequences of the two German TuYV isolates and the full sequence of TuYVaRNA can be found in NCBI GenBank under accession numbers MK450519, MK450520, and MK450521. Raw sequence data are available in the Sequence Read Archive (SRA) under BioProject accession number PRJNA524397 and under BioSample accession numbers SAMN11026350 and SAMN11026351.

## References

[1] Mayo MA 2002 Virology division news: ICTV at the Paris ICV: results of the plenary session and the binomial ballot. Arch Virol 147:2254–2260. doi:10.1007/s007050200052.

[2] Graichen K, Rabenstein F 1996 European isolates of beet western yellows virus (BWYV) from oilseed rape (*Brassica napus* L. ssp. *napus*) are non-pathogenic on sugar beet (*Beta vulgaris* L. var. *altissima*) but represent isolates of turnip yellows virus (TuYV). Z Pflanzenk Pflanzen 103:233–245.

[3] Katul L 1992 Serological and molecular characterization of bean leaf roll virus and faba bean necrotic yellows virus (in German). PhD thesis. University of Göttingen, Göttingen, Germany.

[4] Abraham AD, Menzel W, Lesemann D-E, Varrelmann M, Vetten HJ 2006 Chickpea chlorotic stunt virus: a new polerovirus infecting cool-season food legumes in Ethiopia. Phytopathology 96:437–446. doi:10.1094/PHYTO-96-0437. 18944302

[5] Gaafar Y, Grausgruber-Gröger S, Ziebell H 2016 *Vicia faba, V. sativa* and *Lens culinaris* as new hosts for *Pea necrotic yellow dwarf virus* in Germany and Austria. New Dis Rep 34:28. doi:10.5197/j.2044-0588.2016.034.028.

[6] Abraham AD, Menzel W, Vetten HJ, Saucke H 2007 First report of *Soybean dwarf virus* (genus *Luteovirus*) infecting faba bean and clover in Germany. Plant Dis 91:1059. doi:10.1094/PDIS-91-8-1059B. 30780466

[7] Edgar RC 2004 MUSCLE: multiple sequence alignment with high accuracy and high throughput. Nucleic Acids Res 32:1792–1797. doi:10.1093/nar/gkh340. 15034147 PMC390337

[8] Adams IP, Skelton A, Macarthur R, Hodges T, Hinds H, Flint L, Nath PD, Boonham N, Fox A 2014 *Carrot yellow leaf virus* is associated with carrot internal necrosis. PLoS One 9:e109125. doi:10.1371/journal.pone.0109125. 25365290 PMC4218861

[9] Briddon RW, Ghabrial S, Lin NS, Palukaitis P, Scholthof KBG, Vetten HJ 2012 Satellites and other virus-dependent nucleic acids, p 1211–1219. *In* King AMQ, Lefkowitz E, Adams MJ, Carstens EB (ed), Virus taxonomy: ninth report of the International Committee on Taxonomy of Viruses. Elsevier Academic Press, Oxford, United Kingdom.

[10] Chin LS, Foster JL, Falk BW 1993 The beet western yellows virus ST9-associated RNA shares structural and nucleotide sequence homology with carmo-like viruses. Virologie 192:473. doi:10.1006/viro.1993.1063. 8421895

